# Association between physical activity and the utilization of general practitioners in different age groups

**DOI:** 10.1007/s00508-019-1503-8

**Published:** 2019-05-10

**Authors:** Thomas E. Dorner, Julia Wilfinger, Kathryn Hoffman, Christian Lackinger

**Affiliations:** 1 Department of Social and Preventive Medicine, Centre for Public Health, Medical University of Vienna, Kinderspitalgasse 15/1, 1090 Vienna, Austria; 2 Department for Health Promotion and Prevention, SPORTUNION Austria, Falkestraße 1, 1010 Vienna, Austria; 3 Department of General Practice and Family Medicine, Centre for Public Health, Medical University of Vienna, Kinderspitalgasse 15/I, 1090 Vienna, Austria

**Keywords:** Health enhancing physical activity, EHIS-PAQ, Primary health care, Age, Strength training

## Abstract

**Background:**

Physical activity (PA) is an important tool in health promotion, prevention, curation, and rehabilitation and should be part of general practitioners (GP) consultations. For tailoring GP’s service it is important to know the PA habits of the clients.

**Methods:**

Data from the Austrian Health Interview Survey 2014 with 15,770 subjects were analyzed. The association between PA, measured with the Physical Activity Questionnaire of the European Health Intervies Survey (EHIS-PAQ) and having visited a GP within the last 4 weeks was examined in different age groups (15–29, 30–64, and 65+ years). In multivariate analyses we adjusted for sociodemographic and health-related variables (body mass index, 17 chronic diseases, and the use of medication).

**Results:**

In subjects aged 15–29 years and 30–64 years fulfilling aerobic PA recommendations was significantly associated with a lower chance of having consulted the GP with unadjusted OR (95% CI) 0.82 (0.70–0.96) and 0.90 (0.82–0.99), respectively, whereas work-related PA was associated with a higher chance, with OR 1.21 (1.03–1.42) and 1.10 (1.00–1.20), respectively. Adjusting for sociodemographic and health-related factors led to loss of significance. In subjects aged 30–64 years, muscle strengthening PA was associated with a higher chance for GP consultation with OR 1.12 (1.00–1.24) in the fully adjusted model. In subjects aged 65+ years, PA was associated with a lower chance of having visited the GP with OR 0.74 (0.64–0.86) and 0.83 (0.71–0.97) for work related PA and total PA, respectively, in the fully adjusted model.

**Conclusion:**

The association of PA and GP consultation is dependent on age and type of PA, and partly mediated by sociodemographic and health-related factors.

## Introduction

Physical activity (PA) is an important tool in promoting, maintaining, and recreating health. Further, PA is crucial in various dimension of medical care, from health promotion [1–3], prevention of disease and other adverse outcomes [1, 2, 4], curing of chronic diseases [2], such as obesity [5], type 2 diabetes mellitus [6] and hypertension [7] and medical and social rehabilitation [8, 9]. Undertaking PA is important in all age groups, irrespective of medical conditions, domains of PA (e.g. leisure time PA, active transport, domestic PA, and PA during work), and components (e.g. endurance activities or strength training) and should ideally be combined to achieve the highest effect on health [1, 10, 11].

Primary health care is an appropriate setting to promote a healthy life style, and especially visits to general practitioners (GP) are an important opportunity for life style counselling. The World Health Organization (WHO) proposed that routine contact with health service staff should involve support for individuals to help initiate and maintain healthy behavior as well as give advice on the benefits of PA [2]. In fact, one of the most important factors that increase the amount of PA is the recommendation by a doctor [12], especially a GP [13].

For patients with complex care needs, integrated care has been shown to provide multiple benefits for patients with (severe) health hazards. Comprehensive care means treatment of disease and comorbidities, includes life style intervention, such as regular participation in health-enhancing physical activities, and takes personal, biological, mental as well as social health resources and health barriers into account. Integrated care, a tool for multidisciplinary and multiprofessional teamwork towards optimal patient management has been shown to lead to many positive outcomes, such as better patient management, better adherence to treatment guidelines, better coordination in a fragmented health care system, reduction of hospitalization and health care expenditure and improved quality of life, especially when implemented in the treatment of diseases with complex treatment pathways [14, 15]. In Austria, the primary health care sector is currently under reconstruction. Although there is still a long way to go [16], the aim of rebuilding the primary care in Austria is to integrate various dimensions of care (from health promotion to rehabilitation) into a more multidisciplinary stetting [17], and therefore life style management generally and particularly promoting PA is considered to have an elevated role in future primary care.

The aim of the study was to assess the amount and type of PA in subjects with different amounts of consultation of their GPs in various age groups. Furthermore, the study aimed to analyze whether sociodemographic variables and health status influence the association between PA and consultation of the GP.

## Methods

### Dataset

Data from the Austrian Health Interview Survey (ATHIS), wave 2 in 2014 were analyzed. The ATHIS is a cross-sectional study, based on the European Health Interview Survey (EHIS) and was conducted in 17 European countries [18–20]. The survey focuses on health status, health determinants, health care utilization, and sociodemographic and socioeconomic background characteristics [21]. The ATHIS was carried out from October 2013 to June 2015, by Statistics Austria, primarily via computer-assisted telephone interviewing (CATI). Additionally, some questions, including the questions on PA, were excluded from the CATI survey and participants were asked to fill in a paper questionnaire and return that via mail. The sample was stratified by region, and for each Austrian NUTS-3 region (nomenclature des unités territoriales statistiques) a sample size of 462 subjects (Vienna regions: 560 subjects) was aimed at, yielding a gross sample size of 38,768 subjects. Participants were approached in the frame of the microcensus and via telephone asked to participate in the ATHIS. Of these, 21,343 subjects initially refused to participate. Another 1594 subjects who initially declared their interest to participate, could not be reached or refused the telephone interview, 25 subjects did not finish the interview, and the data of 35 subjects was insufficient. Thus, a net sample of 15,771 subjects were included in the survey, yielding a response rate of 40.7%. Response rate for the paper-based questionnaire was 93%. To increase the response rate, subjects were repeatedly sent reminders and handed out a gift voucher as an incentive.

Data were weighted according to geographic region, age, sex, family situation, migration background, and education level. Missing values were imputated. For the analysis 3 different age groups were formed (15–29 years; 30–64 years; and 65 years and over).

### GP visits

For primary health care utilization people were asked about their last GP visit and could choose between 3 options “last GP visit was less than 12 months ago”; “last GP visit was 12 or more months ago” or “I never visited a GP”. Participants who chose “last GP visit was less than 12 months ago” were asked about the number of GP visits within the last 4 weeks. The GP visits included seeing the doctor in the office, home visits and medical telephone consultations. Arranging an appointment and accompanying a child or another family member did not count as a visit. Participants were further divided into two groups: those who visited their GP in the last 4 weeks once or more and those who did not.

### Physical activity

For the assessment of PA, the PA Questionnaire of the EHIS (EHIS-PAQ) was used [22], which is based on the Global Physical Activity Questionnaire (GPAQ) [23]. This 8‑item questionnaire covers physical activity during work, transportation, aerobic leisure time activity and muscle strengthening during a typical week. Only activities lasting for longer than 10 min were considered, according to recommendations for health enhancing PA [1, 11]. Based on these items, five different binary PA indicators were formed, according to the proposal by the authors of the EHIS-PAQ [22]: (1) work-related PA (WRPA) measures the percentage of subjects being mostly physically active when working; (2) transport-related PA (TRPA) summarizes the minutes per week spent with walking and transport-related bicycling. For formation of the indicator metabolic equivalent (MET) minutes per week were formed by multiplying the minutes per week for walking with 3.3 METs and for cycling with 6 METs. The upper quintile of the MET minutes per week in the respective age groups was used to dichotomize this indicator; (3) compliance with the recommendations for health-enhancing PA (HEPA) was computed by adding up the minutes per week spent with cycling and with aerobic physical activities or sports. Since international and national guidelines for HEPA indicate the performance of at least 150 min as moderate intensity PA per week [1, 11], the indicator was dichotomized as fulfilling this criteria or not; (4) the indicator for muscle strengthening PA (MSPA) guideline compliance was assessed as performing at least twice a week muscle strengthening activities. This is also in line with international and national recommendations [1, 11]; (5) the indicator total PA (TPA) was calculated as fulfilling either the guidelines for HEPA, or being labelled as individual being mostly physically active when working. The EHIS-PAQ has shown good reliability and validity [24].

### Sociodemographic data

Participants’ sex was documented and they were asked for their age. Education levels of the participants was split into three levels: primary education (compulsory school), secondary education (apprenticeship school, professional/commercial school and high school) and tertiary education (university). State of employment was classified into four groups: employed, unemployed, retired, and others (housewife/houseman, students, people unable to work, persons in military service, and people in parental leave).

### Anthropometry

People were asked about their body height and their body weight without shoes and clothes. The body mass index (BMI) was calculated with the formula:

$$BMI=\frac{\textit{body weight in }\mathrm{kg}}{\left(\textit{body height in }\mathrm{m}\right)2}.$$

According to the WHO classification adults were divided into BMI groups:

- Underweight = BMI < 18.5 kg/m^2^
- Normal weight = BMI 18.5–24.9 kg/m^2^
- Overweight = BMI 25.0–29.9 kg/m^2^
- Obesity = BMI ≥ 30 kg/m^2^

### Chronic diseases

Participants were asked whether or not they suffered from chronic diseases and 17 specific chronic diseases were requested and used for the analysis. Those diseases were: bronchial asthma, chronic obstructive pulmonary disease, heart attack or chronic condition after heart attack, coronary heart diseases or angina pectoris, hypertension, stroke or chronic condition after stroke, osteoarthritis, diabetes mellitus, chronic back pain, chronic neck pain, allergy (coryza, food, dermatitis), hepatic cirrhosis, urinary incontinence, kidney disease or renal insufficiency, depression, chronic headache and gastric or duodenal ulcers.

### Medication

Participants were asked if they had taken any prescribed medication in the past 2 weeks. Birth control was excluded; homeopathic supplements, vitamins, minerals, sleeping pills, salves and cough syrup were included.

### Statistical analysis

For descriptive statistics, categorical variables are presented as percentages. Bivariate analyses were performed by means of cross-tabulations and group differences were assessed with the χ^2^-test. Metric variables are presented as means with standard deviation (SD), and univariate ANOVA was performed. Stepwise binary logistic regression analyses were performed to determine the association between PA-related factors and utilization of the GP. Results are presented as odds ratios (OR) and 95% confidence intervals (95% CI). In all regression analyses, attendance at least once at the GP in the last 4 weeks was defined as dependent variable. Stepwise adjustment was carried out for potential confounders due to the known association of various sociodemographic and health-related parameters with PA on the one side and health care utilization on the other side. The first model represents unadjusted analysis. The second model is adjusted for the sociodemographic variables age, sex, employment status, and educational level. The third model is additionally adjusted for health-related factors, such as BMI categories, each of 17 different chronic diseases, and the use of prescribed medication. All parameters except age were used as categorical variables in the models. There was a significant interaction between age group and PA on the GP consultation regarding WRPA (*P* < 0.001), MSPA (*P* = 0.040), and TPA (*P* = 0.004). Therefore, all regression analyses are stratified by age group. Calculations were performed using SPSS Statistics 22 and the statistical significance was set at *p* < 0.05.

### Results

In the general population aged 15 years and older, 47.7% were mostly physically active when working, while 40.1% were not physically active when working and 12.2% were not working. Of the subjects 50.1% fulfilled the minimum requirements for aerobic HEPA of at least 150 min per week moderate intensity PA or cycling, and 32.1% fulfilled the minimum requirements for muscle strengthening PA of at least twice per week. Of the general population 74.1% were either mostly physically active when working or complying with HEPA guidelines. Differences in sociodemographic, health-related, and PA-related characteristics between the age groups are shown in Table 1. With age the proportion of female subjects increased and higher education level was linked to younger age. In the youngest age group most subjects were gainfully employed or had other employment, in the middle age group most subjects were gainfully employed and in the highest age group most subjects were retired. Prevalence of obesity, prevalence of almost all of the assessed chronic diseases, and the proportion of subjects taking prescribed medication increased with age group. The proportion of subjects who fulfilled the minimum recommendation for aerobic HEPA, and for TPA decreased with age, while subjects in the middle age group were those with the highest proportion who were mostly physically active when working, and subjects of the youngest age group were those with the highest proportion complying with MSPA guidelines. The proportion of subjects who consulted their GP within the last 4 weeks increased with increasing age group. The mean number of GP visits (among those who visited their GP in the last 4 weeks) was highest in the middle age group, followed by the oldest age group.

**Table 1:** Characteristics of the sample in different age groups in percent

	Age group 1 (15–29 years)*N* = 3388	Age group 2 (30–64 years)*N* = 9074	Age group 3 (65+ years)*N* = 3308	*P*-value
Physical activity				
WRPA (mostly physically active when working)	38.8	48.4	44.6	<0.001
TRPA (upper age group^a^ specific quintiles of MET minutes/week spent with walking and cycling)	22.7	20.0	19.6	–
HEPA guidelines compliance (at least 150 min per week moderate PA or cycling)	57.5	49.0	45.3	<0.001
MSPA guidelines compliance (at least twice per week muscle-strengthening activity)	43.7	29.5	32.9	<0.001
TPA (mostly physically active when working OR complying with HEPA guidelines)	75.6	74.7	65.5	<0.001
Sex %				
Female	49.2	50.3	56.5	0.001
State of employment %				
Employed	49.7	72.0	1.1	<0.001
Unemployed	7.1	6.1	0.3	
Retired	0.1	13.1	94.2	
Others	43.1	8.8	4.5	
Education level %				
Primary education	29.3	14.9	35.4	<0.001
Secondary education	32.7	54.5	47.9	
Tertiary education	38.1	30.6	16.7	
BMI categories %				
Underweight	6.4	1.8	1.8	<0.001
Normal weight	67.3	48.7	37.5	
Overweight	19.1	34.3	41.1	
Obesity	7.1	15.1	19.6	
Chronic disease %				
Bronchial asthma	3.1	4.2	6.2	<0.001
Chronic obstructive pulmonary disease	1.4	3.6	8.6	<0.001
Heart attack or chronic condition after heart attack	0.0	0.6	2.9	<0.001
Coronary heart diseases or angina pectoris	0.5	1.1	6.9	<0.001
Hypertension	2.4	18.2	48.3	<0.001
Stroke or chronic condition after stroke	0.1	0.6	2.1	<0.001
Osteoarthritis	0.5	9.4	30.8	<0.001
Diabetes mellitus	0.7	3.5	13.3	<0.001
Chronic back pain	9.2	25.0	38.1	<0.001
Chronic neck pain	6.5	20.5	25.3	<0.001
Allergy (coryza, food, dermatitis)	29.2	24.9	17.0	<0.001
Hepatic cirrhosis	0.3	0.2	0.3	0.360
Urinary incontinence	0.1	1.7	12.6	<0.001
Kidney disease or renal insufficiency	0.4	0.9	4.1	<0.001
Depression	3.2	8.1	11.1	<0.001
Chronic headache	7.8	7.1	4.7	<0.001
Gastric or duodenal ulcers	2.5	2.4	2.6	0.858
Prescribed medication in last 2 weeks %	22.4	46.5	84.1	<0.001
Visited GP within the last 4 weeks %	23.2	29.3	42.7	<0.001
Mean number (SD) of GP visits in those who visited GP in last 4 weeks	1.49 (1.10)	1.66 (1.66)	1.60 (1.43)	0.13

*WRPA* work-related physical activity, *TRPA* transport-related physical activity, *HEPA* health-enhancing physical activity, *MSPA* muscle strengthening physical activity, *TPA* total physical activity, *BMI* body mass index, *GP* general practitioner

*due to the fact that age specific quintiles were used no *P*-value has been computed

Table 2 shows differences in sociodemographic, health-related, and PA-related characteristics between those who visited vs. did not visit their GP within the last 4 weeks. Attendees were older, more often female, more often retired or unemployed, and had a lower education level. Prevalence of obesity, and chronic diseases, as well as the proportion of subjects taking prescribed medication was higher in attendees. The proportion of subjects in the upper quintile for TRPA and those who fulfilled the minimum recommendations for aerobic HEPA, for MSPA, and for total TPA was lower in subjects who visited their GP within the last 4 weeks. There was no difference in the proportion of subjects being mostly physically active when working.

**Table 2:** Differences in characteristics in those who did not visit the general practitioner (GP) in the last 4 weeks and patients who visited the GP in the last 4 weeks

	Attendees *n* = 4856	Non-attendees*n* = 10,913	*P*-value
*Physical activity*			
WRPA (mostly physically active when working)	45.5	45.6	0.923
TRPA (upper age group specific quintiles of MET minutes/week spent with walking and cycling)	19.5	20.9	0.037
HEPA guidelines compliance (at least 150 min per week moderate PA or cycling)	46.4	51.7	<0.001
MSPA guidelines compliance (at least twice per week muscle-strengthening activity)	32.0	33.8	0.022
TPA (mostly physically active when working OR complying with HEPA guidelines)	70.5	74.1	<0.001
*Age group %*			
15–29 years	16.2	23.8	<0.001
30–64 years	54.7	58.8	
65+ years	29.1	17.4	
*Sex %*			
Female	54.6	49.9	<0.001
State of employment %			
Employed	43.6	56.2	<0.001
Unemployed	6.2	4.7	
Retired	37.2	22.9	
Others	13.0	16.3	
*Education level %*			
Primary education	26.1	20.5	<0.001
Secondary education	50.6	47.5	
Tertiary education	23.3	32.0	
*BMI categories %*			
Underweight	2.3	3.0	<0.001
Normal weight	44.6	52.9	
Overweight	33.5	32.0	
Obesity	19.6	12.0	
*Chronic disease %*			
Bronchial asthma	6.7	3.4	<0.001
Chronic obstructive pulmonary disease	6.9	3.9	<0.001
Heart attack or chronic condition after heart attack	1.8	0.6	<0.001
Coronary heart diseases or angina pectoris	4.6	1.1	<0.001
Hypertension	33.2	15.8	<0.001
Stroke or chronic condition after stroke	1.8	0.4	<0.001
Osteoarthritis	19.3	8.7	<0.001
Diabetes mellitus	9.0	3.1	<0.001
Chronic back pain	35.1	19.6	<0.001
Chronic neck pain	26.9	14.8	<0.001
Allergy (coryza, food, dermatitis)	27.1	22.9	<0.001
Hepatic cirrhosis	0.3	0.2	0.198
Urinary incontinence	6.4	2.4	<0.001
Kidney disease or renal insufficiency	2.7	0.9	<0.001
Depression	13.4	5.1	<0.001
Chronic headache	9.5	5.5	<0.001
Gastric or duodenal ulcers	3.5	2.1	<0.001
Prescribed medication in last two weeks %	75.3	37.6	<0.001

*WRPA* Work related physical activity, *TRPA* Transport related physical activity, *HEPA* Health enhancing physical activity, *MSPA* Muscle-strengthening physical activity, *TPA* Total physical activity, *BMI* body mass index

In subjects aged 15–29 years, those mostly physically active when working had a significantly higher chance of having visited their GP in the last 4 weeks, while those fulfilling the minimum recommendations for aerobic HEPA had a significantly lower chance of having visited their GP. These associations were not significant any more when adjusted for sociodemographic and health-related parameters. All other measures of PA showed no significant association with GP consultation in this age group (Table 3).

**Table 3:** Association between physical activity (PA) and general practitioner (GP) consultation within the last 4 weeks (dependent variable) in subjects aged 15–29 years

	Model I^a^		Model II^b^		Model III^c^	
	OR	95% CI	OR	95% CI	OR	95% CI
WRPA (mostly physically active when working)	1.21	1.03–1.42	1.15	0.96–1.37	1.18	0.98–1.43
TRPA (upper age group specific quintiles of MET minutes/week spent with walking and cycling)	0.88	0.71–1.05	0.94	0.79–1.14	1.00	0.77–1.18
HEPA guidelines compliance (at least 150 min per week moderate PA or cycling)	0.82	0.70–0.96	0.88	0.75–1.04	0.94	0.78–1.12
MSPA guidelines compliance (at least twice per week muscle-strengthening activity)	0.97	0.83–1.14	1.04	0.88–1.22	1.11	0.93–1.32
TPA (mostly physically active when working OR complying with HEPA guidelines)	1.08	0.89–1.30	1.10	0.91–1.33	1.21	0.98–1.49

^a^unadjusted, ^b^adjusted for age, sex, state of employment and education level, ^c^adjusted for age, sex, state of employment, education level, body mass index category, each of 17 different chronic diseases, and prescribed medication

*WRPA* Work related physical activity, *TRPA* Transport related physical activity, *HEPA* Health enhancing physical activity, *MSPA* Muscle-strengthening physical activity, *TPA* Total physical activity

In persons aged 30–64 years, similarly to the youngest age group, those mostly physically active when working had a significantly higher chance of having visited their GP in the last 4 weeks, while those fulfilling the minimum recommendations for aerobic HEPA had a significant lower chance of having visited their GP. Again, these associations were not significant any more when adjusted for sociodemographic and health-related parameters. In the fully adjusted model, persons fulfilling the recommendations for MSPA had a higher chance of having consulted their GP in the last 4 weeks (Table 4).

**Table 4:** Association between physical activity (PA) and general practitioner (GP) consultation within the last 4 weeks (dependent variable) in subjects aged 30–64 years

	Model I^a^		Model II^b^		Model III^c^	
	OR	95% CI	OR	95% CI	OR	95% CI
WRPA (mostly physically active when working)	1.10	1.00–1.20	1.02	0.93–1.12	1.06	0.95–1.17
TRPA (upper quintiles of MET minutes/week spent with walking and cycling)	0.97	0.86–1.08	0.91	0.81–1.02	0.93	0.82–1.06
HEPA guidelines compliance (at least 150 min per week moderate PA or cycling)	0.90	0.82–0.99	0.92	0.84–1.01	1.01	0.91–1.11
MSPA guidelines compliance (at least twice per week muscle-strengthening activity)	1.03	0.94–1.14	1.07	0.97–1.19	1.12	1.00–1.24
TPA (mostly physically active when working OR complying with HEPA guidelines)	0.96	0.87–1.07	0.96	0.86–1.07	1.07	0.95–1.20

^a^unadjusted; ^b^adjusted for age, sex, state of employment and education level; ^c^adjusted for age, sex, state of employment, education level, body mass index category, each of 17 different chronic diseases, and prescribed medication

*WRPA* Work related physical activity, *TRPA* Transport related physical activity, *HEPA* Health enhancing physical activity, *MSPA* Muscle-strengthening physical activity, *TPA* Total physical activity

In the oldest age group (65+ years), those fulfilling the minimum requirements for HEPA, for MSPA, and for TPA, and those mostly physically active when working, had a significant lower chance of having visited their GP in the last 4 weeks. These associations remained stable when adjusted for sociodemographic variables, and in the case of WRPA and TPA, also when adjusted for health-related parameters (Table 5).

**Table 5:** Association between physical activity (PA) and general practitioner (GP) consultation within the last 4 weeks (dependent variable) in subjects aged 65 years and over

	Model I^a^		Model II^b^		Model III^c^	
	OR	95% CI	OR	95% CI	OR	95% CI
WRPA (mostly physically active when working)	0.68	0.59–0.78	0.68	0.59–0.78	0.74	0.64–0.86
TRPA (upper quintiles of MET minutes/week spent with walking and cycling)	0.90	0.75–1.07	0.90	0.75–1.07	1.04	0.86–1.25
HEPA guidelines compliance (at least 150 min per week moderate PA or cycling)	0.73	0.64–0.84	0.76	0.66–0.87	0.92	0.78–1.07
MSPA guidelines compliance (at least twice per week muscle-strengthening activity)	0.77	0.66–0.89	0.78	0.67–0.91	0.87	0.74–1.02
TPA (mostly physically active when working OR complying with HEPA guidelines)	0.66	0.57–0.76	0.68	0.58–0.79	0.83	0.71–0.97

^a^unadjusted; ^b^adjusted for age, sex, state of employment and education level; ^c^adjusted for age, sex, state of employment, education level, body mass index category, each of 17 different chronic diseases, and prescribed medication

*WRPA* Work related physical activity, *TRPA* Transport related physical activity, *HEPA* Health enhancing physical activity, *MSPA* Muscle-strengthening physical activity, *TPA* Total physical activity

## Discussion

This study found in different age groups diverse associations between the amount and type of PA and the consultation of a GP. While in the oldest age groups there was a clear tendency towards negative association (the more physically active, the lower the probability for GP consultations), in the younger age groups there were positive associations (the more physically active, the higher the probability for GP consultations) as well as negative associations, depending on the type of PA. A negative association can be interpreted in the way that PA is protective against developing or being affected by chronic diseases and therefore more physically active persons do not require the health care system and thus do not need to consult their GP that often. A positive association means that those who more often visit their GP have a higher amount of PA. This can be either due to the fact that exercise is part of the treatment of chronic health conditions or due to the fact that PA is recommended as a tool for health promotion by the GP, and persons who more often visit their GP have a higher probability to get a consultation for healthy life style, especially in the absence of chronic medical conditions; the latter under the assumption, that counselling for PA actually changes health behavior and increases PA levels.

A positive association of PA and consulting the GP was found only regarding WRPA in the younger age groups and MSPA in the middle age group. The positive association regarding WRPA is most probably due to the fact that those who are physically active when working are more likely to have lower education and lower socioeconomic status, and this is often associated with adverse psychosocial factors, adverse health behavior, higher health burden, higher probability of chronic diseases [25, 26], and this leads to the higher chance for GP consultation. In fact, adjusting for sociodemographic and heath-related factors led to a loss of significance in the reported association, and therefore these factors are likely to mediate the association between WRPA and GP consultation. In the middle age group MSPA was associated with GP consultation, only in the fully adjusted model. This fact points more in the direction that healthy subjects are more often consulted towards MSPA by their GP for health promotion and prevention, rather than patients with medical conditions are treated with MSPA. Especially in this age group, strength training is associated with many positive health outcomes [27]. In Austria there are free health examinations, usually carried out by the GP, focusing on health promotion and life style consultations, and these are very well accepted in the Austrian population. Although these examinations are intended for healthy subjects without chronic conditions, they are often attended by persons with medical problems [28]. Fulfilling the aerobic recommendations for HEPA, as well as TPA and WRPA were the PA components which were most strongly associated with a lower chance of GP consultation in the highest age group, partly also in the fully adjusted model, which can be interpreted in the way that especially in higher age, fulfilling the minimum requirements for HEPA has a clear preventive function. Interestingly, TRPA was not significantly associated with GP consultation in any age group. In recent PA guidelines, active transportation is highlighted as an important source for overall PA [10]; however, it must be questioned if only increasing TRPA is intensive enough to effectively contribute to HEPA and the treatment of chronic diseases.

The amount of PA was relatively high in both groups, those who consulted their GP in the last 4 weeks and those who did not. About half of the subjects did not fulfil the minimum aerobic recommendations for PA, and about two thirds did not fulfil the minimum recommendations for strength training. When compared to other counties, the inactivity rate of subjects in primary care in this survey was in the middle: A Spanish survey reported an inactivity rate of 63% of patients 20–80 years old [29], whereas a study in the Netherlands showed an inactivity rate of 38% of patients aged 30–50 years [30].

In this study, the amount of PA did not differ that much between the age groups as expected by intuition; however, there was a tendency towards a decrease of PA amount with increasing age group. On the other hand, the middle age group, the one with most subjects in full employment, was the one with the highest proportion of subjects being physically active when working. Additionally, this was also the age group with the lowest proportion of subjects fulfilling the minimum recommendations for MSPA, but if they fulfilled MSPA recommendations this was mostly associated with having visited their GP in the last 4 weeks. Other Austrian studies have also found a reduction in habitual PA levels that were associated with age [25] or the middle-aged group showed the highest amount of PA [12]. A possible reason for the low level of MSPA in the middle-age group in this study might be that those subjects are mostly full-time employed and they might have no time for more PA. A lack of time is in fact the most common reason indicated by adults for not engaging in regular PA [12].

The results are important for health care planning, especially when reconstructing the primary care level in direction of integrated care. Primary care is the level of care which is among other things characterized by coordination, comprehensiveness and continuity [31, 32]. In the light of these findings PA should be implemented more into primary care in order to achieve comprehensiveness. Although the proportion of subjects fulfilling the aerobic criteria for HEPA is high, this proportion could still be improved, and especially the low proportion of patients who fulfil the minimum requirements for MSPA requires urgent action. PA is a tool with its right in all dimensions of care, from health promotion to rehabilitation, irrespective of the presence or absence of medical conditions. Especially in primary care where continuity of care should be maintained from young to old, and from disease-free to palliation, PA should be considered as an important tool to realize several health objectives. Finally, primary care is the appropriate setting for coordination and means that primary care can be the right place for referral to sports organizations who can implement and maintain regular PA. In fact, such referral schemes are currently under investigation in Austria [33].

It is important to interpret the results of the study in the context of the Austrian health care system. There is no gate-keeping in Austria, meaning that with only few exceptions, every level of care (specialists, hospital clinics, hospitals, university hospitals) can be accessed directly without consulting a GP beforehand. This is especially often performed by younger subjects, persons with higher education, subjects in big cities and patients with a migration background [34]. Therefore, adjusting for sociodemographic factors is crucial for the interpretation of the results. No gate-keeping and low level of coordination in the health care system, also means that PA as treatment for chronic diseases might also be performed in higher levels of the health care system, because some people with chronic conditions simply do not attend the primary care level. Therefore, the aspect of PA as treatment option could be underestimated in this analysis.

The instrument used for PA (EHIS-PAQ) is a relative new instrument, which was introduced in the second wave of the EHIS (and thus in the second wave of AT-HIS). The EHIS-PAQ showed good reliability. Compared with the long form of the International PA Questionnaire (IPAQ), the EHIS-PAQ showed moderate to strong coefficients for validity. The EHIS-PAQ, however, underestimated moderate to vigorous PA, when compared to objectively measured PA [24]. The advantage of the EHIS-PAQ is that it measures PA in different domains, such as active transport, PA during work, and components, such as endurance and strength activities; however, recent studies have shown that work-related PA is not necessarily associated with a gain in health [35]. A further limitation of the EHIS-PAQ is that TRPA is reported only as the upper quintile, so in all analyzed groups, approximately 20% are labelled as being highly active with transportation, due to this methodology. This makes comparisons of subgroups difficult.

A strength of the study is the large sample size with more than 15,000 subjects and that the study deals with a population-based sample and not merely with patients in a clinical setting. This has the advantage that the vast majority of subjects, which are not patients in the health care setting for any reason could be analyzed, and this is very important for the research question. Another strength is that the national investigation is based on an international survey and therefore results can be compared on a European level. A clear limitation is also that the study is based on a cross-sectional survey, therefore no conclusions can be drawn about causes and consequences, or about the timeline between the two main factors analyzed, amount of PA, and consultation of the GP. Another limitation is that PA is recorded only as self-reported, and this usually leads to over-reporting of the amount of PA.

The conclusion that can be clearly drawn from this study is that the association between the habitual level of PA and the consultation of GPs is dependent on the age group and also dependent on the type of PA. There is a tendency that PA is associated with less GP consultation, most probably due to the preventive effect of PA towards developing chronic diseases. This is especially pronounced in higher age groups. Furthermore, predominately in younger age groups, some kinds of PA are associated with higher consultations of the GP, possible as result of the fact that PA is part of the treatment of chronic diseases, or due to the fact that PA is recommended as a tool for health promotion by the GP. Overall, the results highlight the importance of PA in primary care and its importance in various dimensions of care, such as health promotion, prevention, curing, and rehabilitation, and therefore PA in primary care is an important tool towards patient centered and integrated care.

## References

[1] Physical Activity Guidelines Advisory Committee (2008). Physical activity guidlines advisory committee report 2008.

[2] World Health Organization (2010). Global recommendations on physical activity for health.

[3] Physical Activity Guidelines Advisory Committee (2018). 2018 physical activity guidelines advisory committee scientific report.

[4] Haider S, Grabovac I, Dorner TE (2018). Fulfillment of physical activity guidelines in the general population and frailty status in the elderly population : A correlation study of data from 11 European countries. Wien Klin Wochenschr.

[5] Uerlich MF, Yumuk V, Finer N, Basdevant A, Visscher TL (2016). Obesity management in Europe: current status and objectives for the future. Obes Facts.

[6] Eder S, Leierer J, Kerschbaum J (2018). Guidelines and clinical practice at the primary level of healthcare in patients with type 2 diabetes mellitus with and without kidney disease in five European countries. Diab Vasc Dis Res.

[7] Williams B, Mancia G, Spiering W (2018). 2018 ESC/ESH Guidelines for the management of arterial hypertension. Eur Heart J.

[8] Lackinger C, Wilfinger J, Mayerhofer J (2017). Adherence to and effects on physical function parameters of a community-based standardised exercise programme for overweight or obese patients carried out by local sports clubs. Public Health.

[9] Niebauer J, Mayr K, Harpf H (2014). Long-term effects of outpatient cardiac rehabilitation in Austria: a nationwide registry. Wien Klin Wochenschr.

[10] U.S. Department of Health and Human Services (2018). Physical activity guidelines for Americans.

[11] Titze S, Ring-Dimitriou S, Schober PH (2010). Österreichische Empfehlungen für gesundheitswirksame Bewegung.

[12] Wepner F, Hahne J, Machacek P, Holzapfel J, Friedrich M (2009). Motivation for physical activity—a survey in a Central European state. Wien Klin Wochenschr.

[13] Douglas F, Torrance N, van Teijlingen E, Meloni S, Kerr A (2006). Primary care staff’s views and experiences related to routinely advising patients about physical activity. A questionnaire survey. BMC Public Health.

[14] Allen D, Gillen E, Rixson L (2009). Systematic review of the effectiveness of integrated care pathways: what works, for whom, in which circumstances?. Int J Evid Based Healthc.

[15] Sun X, Tang W, Ye T (2014). Integrated care: a comprehensive bibliometric analysis and literature review. Int J Integr Care.

[16] Hoffmann K, Wojczewski S, Aarendonk D (2017). No common understanding of profession terms utilized in health services research : An add-on qualitative study in the context of the QUALICOPC project in Austria. Wien Klin Wochenschr.

[17] Hofmarcher MM, Mayer S, Peric N, Dorner TE, Braithwaite J (2018). Primary healthcare centers: a silver bullet?. Healthcare systems. Future predictions for global care.

[18] Aromaa A, Koponen P, Tafforeau J, Vermeire C, Group HHC (2003). Evaluation of Health Interview Surveys and Health Examination Surveys in the European Union. Eur J Public Health.

[19] European Commission (2009). The components of the European health survey system.

[20] Eurostat (2013). European Health Interview Survey (EHIS wave 2). Methodological manual.

[21] Statistik Austria (2014). Die Österreichische Gesundheitsbefragung 2014 (ATHIS).

[22] Finger JD, Tafforeau J, Gisle L (2015). Development of the European Health Interview Survey—Physical Activity Questionnaire (EHIS-PAQ) to monitor physical activity in the European Union. Arch Public Health.

[23] World Health Organization (2012). Global physical activity questionnaire (GPAQ) analysis guide.

[24] Baumeister SE, Ricci C, Kohler S (2016). Physical activity surveillance in the European Union: reliability and validity of the European Health Interview Survey-Physical Activity Questionnaire (EHIS-PAQ). Int J Behav Nutr Phys Act.

[25] Dorner TE, Stronegger WJ, Hoffmann K, Stein KV, Niederkrotenthaler T (2013). Socio-economic determinants of health behaviours across age groups: results of a cross-sectional survey. Wien Klin Wochenschr.

[26] Dorner TE, Stronegger WJ, Rebhandl E, Rieder A, Freidl W (2010). The relationship between various psychosocial factors and physical symptoms reported during primary-care health examinations. Wien Klin Wochenschr.

[27] Grabovac I, Haider S, Winzer E (2018). Changes in health parameters in older lay volunteers who delivered a lifestyle-based program to frail older people at home. Wien Klin Wochenschr.

[28] Brunner-Ziegler S, Rieder A, Stein KV (2013). Predictors of participation in preventive health examinations in Austria. BMC Public Health.

[29] Grandes G, Sanchez A, Montoya I (2011). Two-year longitudinal analysis of a cluster randomized trial of physical activity promotion by general practitioners. PLoS ONE.

[30] Lakerveld J, Bot SD, Chinapaw MJ (2013). Motivational interviewing and problem solving treatment to reduce type 2 diabetes and cardiovascular disease risk in real life: a randomized controlled trial. Int J Behav Nutr Phys Act.

[31] Dorner TE, von Mittelstaedt G (2018). Primary care in the German speeking area. Gesundheitswesen.

[32] Starfield B (1992). Primary care: concept, evaluation, and policy.

[33] Lackinger C, Strehn A, Dorner TE, Niebauer J, Titze S (2015). Health resorts as gateways for regional, standardised, sports club based exercise programmes to increase the weekly time of moderate- to vigorous-intensity physical activity: study protocol. BMC Public Health.

[34] Hoffmann K, Stein KV, Maier M, Rieder A, Dorner TE (2013). Access points to the different levels of health care and demographic predictors in a country without a gatekeeping system. Results of a cross-sectional study from Austria. Eur J Public Health.

[35] Holtermann A, Krause N, van der Beek AJ, Straker L (2018). The physical activity paradox: six reasons why occupational physical activity (OPA) does not confer the cardiovascular health benefits that leisure time physical activity does. Br J Sports Med.

